# Observational study of surgical resection in small non-functional pancreatic neuroendocrine tumors: AS SEER-based study

**DOI:** 10.1038/s41598-023-39980-z

**Published:** 2023-08-07

**Authors:** Jiajing Lin, Heguang Huang

**Affiliations:** Department of General Surgery, Fujian Medical University Union Hospital, #29 Xinquan Road, Fuzhou, 350001 People’s Republic of China

**Keywords:** Cancer therapy, Cancer therapeutic resistance

## Abstract

The potential benefits of surgical resection for small non-functional pancreatic neuroendocrine tumors (NF-PNETs) in terms of survival remain uncertain. This study aimed to evaluate the impact of surgical treatment on patients with NF-PNETs. Using SEER data, we identified 1102 patients from 2004 to 2015 with well and moderately differentiated pancreatic neuroendocrine tumors (PNETs). The associations between continuous variables and receipt of surgery were assessed using Wilcoxon rank-sum tests. Kaplan–Meier survival curves for OS were compared using the log-rank test. We compared outcomes in patients who received surgical resection with those in patients who did not, using a univariable Cox model with inverse probability weighting according to the propensity score and propensity-score matching. Among the cohort of 1102 patients, a majority of 965 individuals (87%) underwent surgical intervention. Upon conducting univariate analysis, we observed that surgical treatment significantly prolonged patients' survival [HR = 0.41, 95% CI [0.26–0.65] *P* < 0.001]. However, the old [HR = 3.27, 95% CI (2.24–4.76), *P* 0.001], male gender [HR = 1.82, 95% CI (1.23–2.68), *P* = 0.003], and moderately well-differentiated factors [HR = 1.71, 95% CI (1.04–2.80), *P* = 0.034] were found to potentially decrease patients' survival time. In the multivariate analysis, male gender [HR = 1.73, 95% CI (1.15–2.61), *P* = 0.009] and the old factor [HR = 3.52, 95% CI (2.33–5.31), *P* < 0.001] emerged as influential predictors with higher hazard ratios. Notably, surgical treatment remained a significant factor associated with improved overall survival [HR = 0.53, 95% CI (0.33–0.84), *P* = 0.007]. Propensity-score matching and inverse probability weighting were employed as analytical techniques. The univariate analysis results showed favorable outcomes in the weight group [HR = 0.48, 95% CI (0.29–0.78), *P* = 0.003] and matched group [HR = 0.44, 95% CI (0.22–0.85), *P* = 0.015], respectively. Survival analysis further confirmed that surgical treatment contributed to increased overall survival (log rank, *P* < 0.05) in both the matching and weight groups. Patients diagnosed with small, non-functioning pancreatic neuroendocrine tumors who undergo surgical intervention exhibit improved overall survival (OS) outcomes. Therefore, surgery is strongly recommended for this patient population.

## Introduction

Pancreatic neuroendocrine tumors (PNETs) constitute a heterogeneous category of infrequent neoplasms that arise within the diffuse neuroendocrine system of the pancreas^1^. These tumors possess the capacity to undergo a gradual metamorphosis from a benign to a malignant state. The prevalence rates of PNETs have escalated to an impressive 10% among all pancreatic tumors^2^. Presently, as the National Comprehensive Cancer Network (NCCN) guidelines (V1.2015), the surgical excision of PNETs exceeding 2 cm in size is advocated^3^. Nevertheless, a contentious debate persists concerning the role of surgery in the management of small non-functional pancreatic neuroendocrine tumors (NF-PNETs), particularly those characterized by a well-differentiated nature^4^.

The Chinese guidelines for the diagnosis and treatment of pancreatic neuroendocrine tumors propose that when PNETs measure ≤ 2 cm in size, remain localized within the pancreas, exhibit no signs of vascular invasion, and display a proportion of HPF and Ki-67-positive cells of less than or equal to 2%, they can be deemed as benign tumor. Nonetheless, fulfilling this criterion frequently necessitates confirmation through postoperative pathological assessment^5^. However, the attainment of this criterion commonly necessitates the confirmation of postoperative pathology. As a result, preoperative diagnostic methods such as imaging fail to comprehensively identify aggressive tumors measuring less than 2 cm in size.

This outcome contributes to the escalation of uncertainty surrounding the necessity of surgical intervention. Several studies propose that NF-PNETs may not require immediate surgical intervention but rather necessitate diligent surveillance^6–8^. However, it is important that these studies have relatively limited median follow-up durations, thus leaving the long-term outcomes in doubt. Conversely, some research endeavors have demonstrated that surgical intervention augments the overall survival rates of NF-PNETs^8–10^. Although surgical resection remains the exclusive curative modality for NF-PNETs, pancreatic resections pose notable perils in terms of morbidity and mortality. While the risks associated with pancreatic surgery may be deemed justifiable for symptomatic hormone-producing PNETs and larger tumors, the optimal approach to managing NF-PNETs is less straightforward. It is important to recognize that pancreatectomy carries a significant risk of complications and death, and thus, the potential benefits of surgical removal for survival in the case of NF-PNETs remain uncertain.

This study endeavors to investigate whether surgical excision of such tumors can enhance the survival duration of patients afflicted with NF-PNETs. Furthermore, propensity scores were employed to evaluate the impact of other factors on the surgical outcomes.

## Method

### Data source

The data were extracted from the Surveillance, Epidemiology, and End Results (SEER, https://seer.cancer.gov) database using SEER*Stat software Version 8.3.6. Data from patients with Pancreatic neuroendocrine tumors (PNETs) diagnosed in 2004–2015 who had complete information including age, sex, race, primary site, grade, marital status, T stage, N stage, tumor size, histology were included in the study.

The surveillance, epidemiology, and results (SEER) database (https://seer.cancer.gov/seerstat), which contains prospectively collected data on demographics, lesion, the first course of treatment, and survival of all cancer patients from state cancer registries across the United States, was utilized for case extraction.

### Inclusion criteria

1. Diagnosed with PNETs as defined by the International Classification of Disease codes (ICD-O-3): (8150/3: Pancreatic endocrine tumor; 8240/3: Carcinoid tumor; 8246/3: Neuroendocrine carcinoma and 8249/3: Atypical carcinoid tumor) and site code (C25.0-C25.4 and C25.7-C25.9) in 2004–2015. The follow up time was 5 years.
2. Patients with tumors sizes ≤ 2 cm (T1) without regional lymph node involvement (N0) or distant metastasis (M0).
3. Based on grade, patients with tumor moderately well-differentiated and well-differentiated were included
4. Patients had completed follow-ups.

### Exclusion criteria

1. Patients diagnosed by the International Classification of Disease codes (ICD-O-3) not (8150/3: Pancreatic endocrine tumor; 8240/3: Carcinoid tumor; 8246/3: Neuroendocrine carcinoma and 8249/3: Atypical carcinoid tumor) were excluded.
2. Patients with poorly differentiated (grade 3) or undifferentiated/anaplastic (grade 4) tumors were excluded.
3. Patients with multiple cancers and autopsy/death certificate only and missing staging data were also excluded; no patients were found to have missing receipt of surgery data.

### Definition of variables and endpoint

Our analysis involved extracting clinicopathologic variables such as age at diagnosis, race (categorized as white, Black, and other), sex (male or female), tumor differentiation grade (well-differentiated or moderately differentiated), tumor size, marital status (categorized as married, single, or unknown), and primary tumor size (categorized as head, body, tail, or other). The predictor variable was receipt of surgery, defined by codes 25–90 in the RX Sum Surg Prim Site 1998. The primary outcome variable was overall survival (OS). Overall survival was calculated from the date of disease detection until the occurrence of death from any cause.

### Statistical analysis

#### Crude analysis

The basic clinical characteristics of patients in the surgical and non-surgical groups were compared using either the *t* test or chi-square test. The continuous predictor, age, was categorized following an assessment using restricted cubic splines. This approach was employed to relax the assumption of a linear relationship between age and the risks of death^11^. Cox multivariate regression analysis was conducted to examine the association between variables and overall survival (OS), yielding hazard ratios (HR) and 95% confidence intervals. The clinicopathological features of patient survival were assessed through Kaplan–Meier curves and compared using the log-rank test.

#### Propensity-score analyses

To help account for the nonrandomized treatment administration of surgery, we used propensity-score methods to reduce confounding effects. The individual propensities for receiving surgical treatment were estimated using a multivariable cox regression model that included the same covariates as the Cox regression model. Associations between surgery treatment and death were then evaluated by multivariable Cox regression models using two propensity-score methods.

#### Inverse probability weighting

The primary analysis employed inverse probability weighting. In this analysis, the propensity-score model^12^ was utilized to estimate predicted probabilities, which were then used to compute stabilized inverse probability weighting weights^13^. Kaplan–Meier curves and Cox models incorporating the inverse probability weighting weights were utilized in the analysis.

#### Propensity-score matching

We conducted a secondary analysis that used propensity-score matching. Patients in the surgical and non-surgical groups underwent 1:1 Propensity Score Matching (PSM) using the PSM method. In order to alleviate selection bias, confounding factors in non-randomized studies were harmonized in a manner akin to randomization. The matching factors included sex, age, ethnicity, tumor location, TNM stage, and tumor grade. In the propensity-score matching analysis, the greedy matching method was applied to create a matched control sample, the effect value was 0.05.

#### Sensitivity analysis

We used sensitivity analysis in propensity-score matching results. Sensitivity analysis proceeds as follows. First, a series of G values starting 1 is selected. Then, the Wilcoxon signed-rank test (for continuous outcomes) or the McNemar's test (for binary outcomes) is conducted for each G. Lastly, the upper bound of the *P* value of the test statistic is inspected for changes over the series of G values. The treatment effect estimate is considered sensitive if a small increase in G from 1 (no bias to small bias) changes its significance value from significant (e.g., *P* < 0.05) to non-significant (e.g., *P* > 0.05). If statistical significance does not change until a very large G, then the effect estimate is considered very robust to hidden bias^14^. Cox multivariate regression analysis was conducted using the R software (version 3.6.1 R Project for Statistical Computing) to enable an analysis of overall survival (OS). For each analysis, *P* ≤ 0.05 is the threshold for significance.

## Result

### Clinicopathologic characteristics of patients

A total of 1102 patients diagnosed with NF-PNETs were identified between the years 2004 and 2015. The baseline characteristics of these patients are presented in Table 1. Among them, 965 patients (87%) underwent surgical treatment, while 137 patients (13%) did not undergo surgical intervention. A secondary analysis was conducted, employing propensity-score matching. The greedy matching method was employed to generate a matched control sample within the propensity-score matching analysis, as presented in Table 1.

**Table 1:** Characteristics of stage I PNETs (n = 1102).

	Unmatch			Match		
	Surgery	Observation	*P*	Observation	Surgery	*P*
n						
Age (median)	965	137		137	137	
Younger	753 (78.0)	69 (50.4)	0.006	67 (48.9)	69 (50.4)	0.904
Old	212 (22.0)	68 (49.6)		70 (51.1)	68 (49.6)	
Sex (%)						
Female	493 (51.1)	59 (43.1)	0.096	81 (59.1)	59 (43.1)	0.011
Male	472 (48.9)	78 (56.9)		56 (40.9)	78 (56.9)	
Race (%)						
White	748 (77.5)	110 (80.3)	0.750	104 (75.9)	110 (80.3)	0.832
Black	98 (10.2)	14 (10.2)		16 (11.7)	14 (10.2)	
Other	103 (10.7)	12 (8.8)		16 (11.7)	12 (8.8)	
Unknown	16 (1.7)	1 (0.7)		1 (0.7)	1 (0.7)	
Marital (%)						
Marry	645 (66.8)	84 (61.3)	0.385	59 (43.1)	84 (61.3)	0.01
Single	70 (7.3)	10 (7.3)		15 (10.9)	10 (7.3)	
Other	250 (25.9)	43 (31.4)		63 (46.0)	43 (31.4)	
Grade (%)						
Well	864 (89.5)	118 (86.1)	0.294	119 (86.9)	118 (86.1)	1
Moderate	101 (10.5)	19 (13.9)		18 (13.1)	19 (13.9)	
Tumor_size (%)						
≤ 1 cm	298 (30.9)	49 (35.8)	0.292	48 (35.0)	49 (35.8)	1
1–2 cm	667 (69.1)	88 (64.2)		89 (65.0)	88 (64.2)	
Primary tumor size (%)						
Head	210 (21.8)	38 (27.7)	0.004	65 (47.4)	38 (27.7)	0.001
Body and tail	586 (60.7)	63 (46.0)		54 (39.4)	63 (46.0)	
Other	169 (17.5)	36 (26.3)		18 (13.1)	36 (26.3)	

### Crude analysis

We then used univariate and multivariate analysis to evaluate the predictive value of the included variables (Table 2). Using the restricted cubic spline method, we defined older patients as those aged older than 65 years, while younger patients were classified as those aged 65 years and younger (Supplement Fig. 1). In our analysis, we observed that surgical treatment exhibited a protective effect [HR = 0.41, 95% CI (0.26–0.65), *P* < 0.001], indicating an increased overall survival rate. Conversely, factors such as age > 65 [HR = 3.99, 95% CI (2.66–5.98), *P* < 0.001], male gender [HR = 1.82, 95% CI (1.23–2.68), *P* = 0.003], and moderate grade [HR = 1.71, 95% CI (1.04–2.80), *P* = 0.034] were associated with a decrease in the patient's survival time. Furthermore, in the multivariate analysis, male gender [HR = 1.73, 95% CI (1.15–2.61), *P* = 0.009], moderate grade [HR = 0.53, 95% CI (0.33–0.84), *P* = 0.007], and old [HR = 3.52, 95% CI (2.33–5.31), *P* < 0.001] were identified as influential predictors with higher hazard ratios. However, surgical treatment remained a significant factor associated with increased overall survival [HR = 0.53, 95% CI (0.33, 0.84), *P* = 0.007] in the multivariate analysis (Table 2).

**Table 2:** Cox proportional hazards regression model for overall survival.

Characteristic	Univariate		Multivariate	
	HR.CI95	*P*	HR (95% CI)	*P*
Age				
Younger	1.00 [reference]		1.00 [reference]	
Old	3.99 [2.66–5.98]	< 0.001	3.52 [2.33–5.31]	< 0.001
Sex				
Female	1.00 [reference]		1.00 [reference]	
Male	1.82 [1.23–2.68]	0.003	1.73 [1.15–2.61]	0.009
Race (%)				
White	1.00 [reference]		1.00 [reference]	
Black	0.6 [0.28–1.29]	0.192	0.71 [0.32–1.55]	0.387
Other	0.98 [0.52–1.82)]	0.939	1.09 [0.58–2.04]	0.791
Unknown	0 [0–Inf]	0.994	0.00 [0.00–Inf]	0.995
Marital (%)				
Marry	1.00 [reference]		1.00 [reference]	
Single	1.42 [0.73–2.76]	0.299	1.69 [0.85–3.35]	0.132
Other	1.21 [0.8–1.84]	0.373	1.41 [0.91–2.19]	0.122
Grade (%)				
Well	1.00 [reference]		1.00 [reference]	
Moderate	1.71 [1.04–2.8]	0.034	1.66 [1.01–2.73]	0.048
Tumor size (%)				
≤ 1 cm	1.00 [reference]		1.00 [reference]	
1–2 cm	0.71 [0.49–1.04]	0.081	0.80 [0.54–1.18]	0.257
Surgery				
No	1.00 [reference]		1.00 [reference]	
Yes	0.41 [0.26–0.65]	< 0.001	0.53 [0.33–0.84]	0.007
Primary tumor size				
Head	1.00 [reference]		1.00 [reference]	
Body and tail	0.95 [0.59–1.53]	0.840	0.87 [0.54–1.40]	0.556
Other	1.18 [0.68–2.06]	0.551	0.99 [0.56–1.74]	0.961

### Propensity score analysis

**Figure 1:**
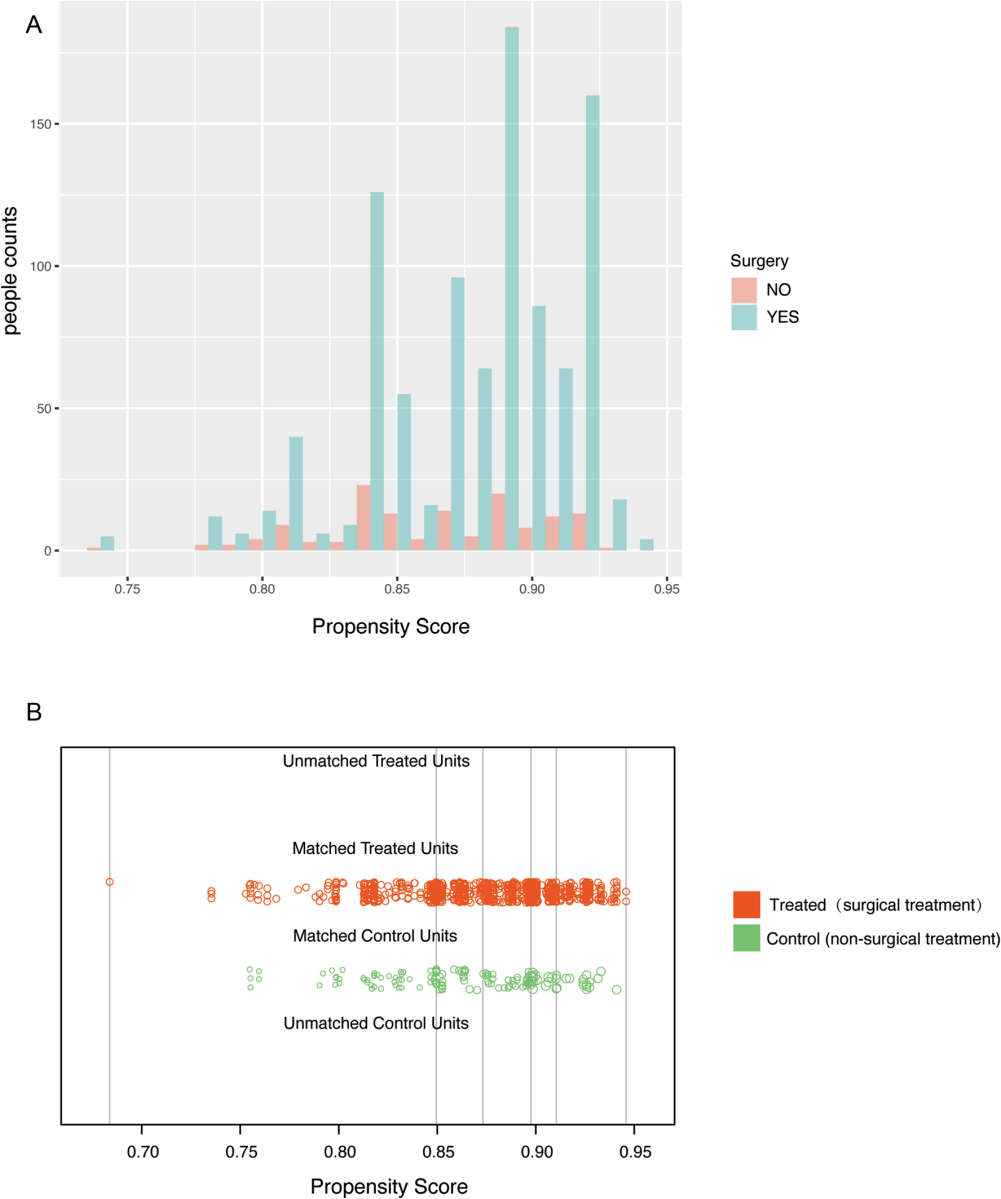
Propensity-score step. (**A**) The propensity score distribution: the frequency distribution of the surgical cohort and the observation cohort, with the surgical group depicted in shades of green and the observation group represented by hues of red. (**B**) Jitter plot of propensity score: each circle symbolizes an individual's propensity score (PS), with the size of the circle indicating their weight. The absence of circles in the highest and lowest strata signifies the absence of unmatched cases within each subgroup.

Propensity scores were employed and the matching variables were visualized. We observed a substantial overlap in the distribution of scores between patients in the surgery group and those in the observation group (Fig. 1A). Following the matching process, each patient's score demonstrated a tendency towards consistency, with no instances of mismatch (Fig. 1B). Univariate and multivariate analysis after matching is shown in Table 3. In univariate analysis, we observed that surgical treatment exhibited a protective effect [HR = 0.44, 95% CI (0.22–0.85), *P* = 0.015]. Conversely, factors such as old [HR = 2.52, 95% CI (1.25–5.08), *P* = 0.01] and male gender [HR = 2.02, 95% CI (1.04–3.91), *P* = 0.037] were associated with a decrease in the patient's survival time. Furthermore, in the multivariate analysis, the old patients [HR = 2.15, 95% CI (1.05–4.41), *P* = 0.037] were identified as influential predictors with higher hazard ratios. However, surgical treatment remained a significant factor associated with increased overall survival (HR = 0.41,95% CI (0.20–0.82), *P* = 0.011) in the multivariate analysis (Table 3). In general, advanced age and the implementation of surgical intervention are predictive factors for patient survival.

**Table 3:** Cox proportional hazards regression model for overall survival after PSM.

Characteristic	Univariate		Multivariate	
	HR.CI95	*P*	HR (95% CI)	*P*
Age				
Younger	1.00 [reference]		1.00 [reference]	
Old	2.52 [1.25–5.08]	0.01	2.15 [1.05–4.41]	0.037
Sex				
Female	1.00 [reference]		1.00 [reference]	
Male	2.02 [1.04–3.91]	0.037	1.69 [0.81–3.52]	0.163
Race (%)				
White	1.00 [reference]		1.00 [reference]	
Black	0.46 [0.11–1.91]	0.282	0.58 [0.14–2.48]	0.465
Other	1.88 [0.78–4.52]	0.159	1.76 [0.69–4.49]	0.238
Unknown	0 [0–Inf]	0.996	0.00 [0.00–Inf]	0.997
Marital (%)				
Marry	1.00 [reference]		1.00 [reference]	
Single	1.09 [0.37–3.18]	0.882	1.45 [0.49–4.32]	0.507
Other	0.98 [0.49–1.93]	0.942	1.37 [0.66–2.86]	0.402
Grade (%)				
Well	1.00 [reference]		1.00 [reference]	
Moderate	1.4 [0.62–3.19]	0.418	1.43 [0.61–3.36]	0.411
Tumor size (%)				
≤ 1 cm	1.00 [reference]		1.00 [reference]	
1–2 cm	0.86 [0.45–1.65]	0.657	0.83 [0.43–1.63]	0.597
Surgery				
No	1.00 [reference]		1.00 [reference]	
Yes	0.44 [0.22–0.85]	0.015	0.41 [0.20–0.82]	0.011
Primary tumor size				
Head	1.00 [reference]		1.00 [reference]	
Body and tail	1.81 [0.88–3.74]	0.108	1.28 [0.59–2.77]	0.527
Other	0.77 [0.27–2.22]	0.631	0.60 [0.20–1.80]	0.359

Given the influence of the propensity score on reducing the patient count in the observation group, we employed the inverse probability weighting method. By augmenting the number of patients in the observation group, we ensured consistency with the number of patients in the surgical group. Following this adjustment, we conducted univariate regression analysis to investigate the impact of surgical factors. In the univariate analysis of surgical treatment, both the weight group [HR = 0.48, 95% CI (0.29–0.78), *P* = 0.003] and matched group [HR = 0.44, 95% CI (0.22–0.85), *P* = 0.015] demonstrated a protective effect, as presented in Table 4. These findings consistently identified surgical treatment as a protective factor.

**Table 4:** Associations between surgical treatment and the death in the crude analysis, univariate analysis, and propensity-score analyses.

Analysis	HR	95% CI	*P*
Crude analysis			
Unmatched	0.41	(0.26–0.65)	< 0.001
Multivariable analysis—hazard ratio (95% CI)			
Unmatched	0.53	(0.33, 0.84)	0.007
Propensity-score analyses—hazard ratio (95% CI)			
Matched	0.44	(0.22–0.85)	0.015
Weighted	0.48	(0.29–0.78)	0.003

### Survival analysis

**Figure 2:**
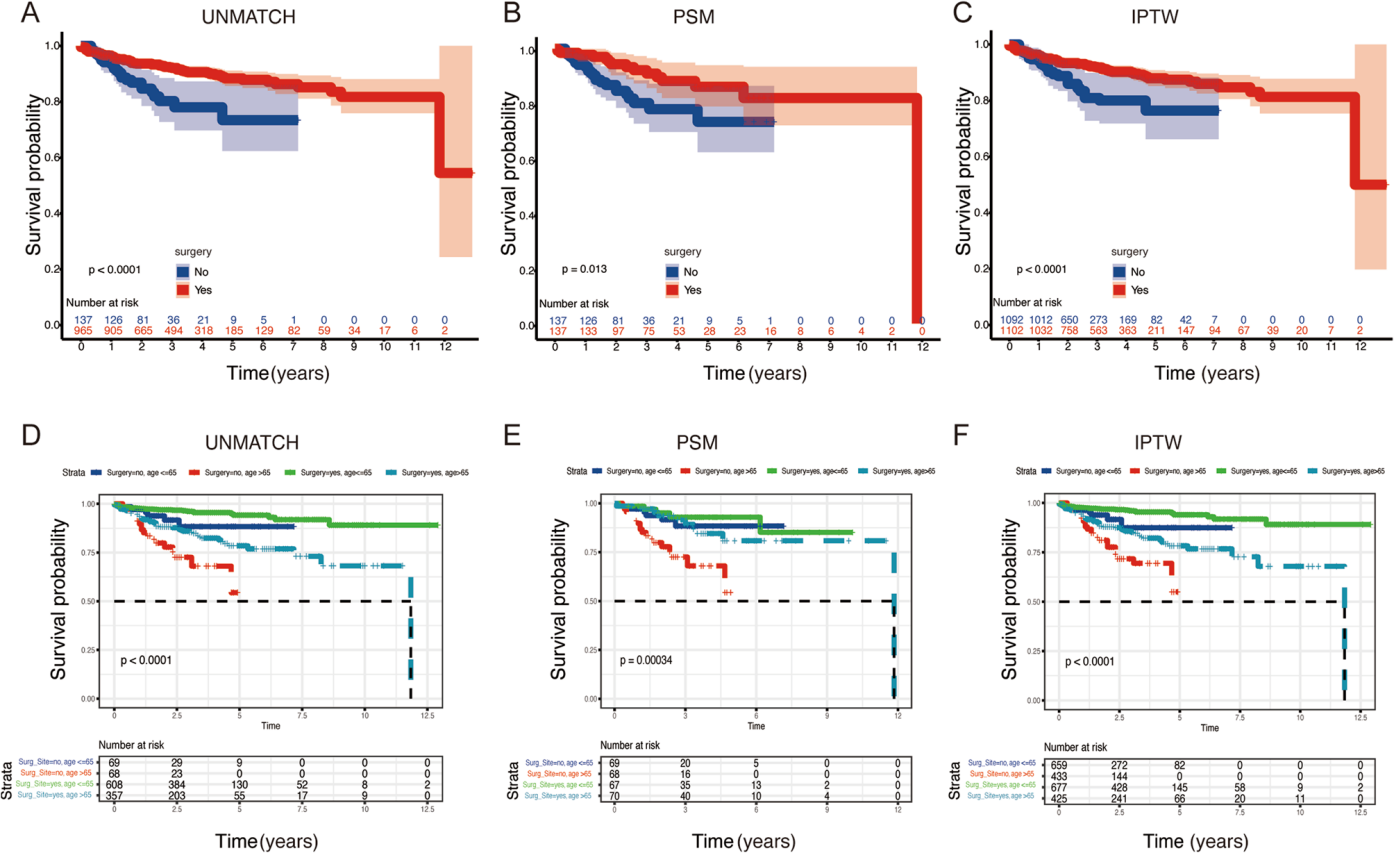
Overall survival analysis of PNETs patients in different method group: (**A**) unmatch; (**B**) propensity-score match; (**C**) inverse probability weighting. Kaplan–Meier estimate of overall survival by subgroup analysis: (**D**) unmatch; (**E**) propensity-score match; (**F**) inverse probability weighting.

The survival analysis which including crude analysis, propensity-score matching analysis and Inverse probability weighting analysis, identified that the surgical treatment could increase the overall time (log-rank, *P* < 0.05) (Fig. 2A–C). In order to evaluate surgical outcomes across different age groups, subgroup analyses were conducted. The overall survival (OS) results are illustrated in Fig. 2D–F. Among the various subgroups analyzed, it was observed that younger individuals who underwent surgery experienced the most significant improvements in survival. Conversely, older individuals who did not undergo surgery exhibited the poorest prognosis in terms of survival.

### Sensitivity analysis

The results displayed in Supplement Table 1 indicate that the treatment effect, in terms of improving survival time, remains statistically significant even under the assumption of no hidden bias (Gamma = 1), as evidenced by the upper bound of the *P* value being less than 0.001. Furthermore, this statistical significance persists even if the treated individuals were up to four times more likely to receive the treatment than their matched counterparts, as indicated by the upper bound of the *P* value being 0.0048 at Gamma = 3. In other words, the treatment effect estimate for this outcome is robust to hidden bias from unmeasured covariates up to Gamma3.

## Discussion

The rarity of small non-functional pancreatic neuroendocrine tumors (NF-PNETs) poses a challenge in accurately assessing their prognosis. Moreover, the treatment of NF-PNETs is complex and lacks consistency, which further contributes to the unpredictability of optimal treatment and prognostic factors. However, the SEER database offers researchers a valuable resource with a large sample size, enabling the identification of factors associated with patient survival. This is particularly advantageous when studying rare tumors, especially when utilizing surgical data. The present study utilized propensity score matching (PSM) and multivariate regression analysis, which revealed that surgery positively impacted overall survival (OS) in patients with SN-PNETs.

Some guidelines suggested surgery as the default management strategy for small NF-PNETs^15,16^, and several studies have demonstrated the benefit of surgery on OS for small, non-functional PNETs^4,9^. For example, a population-based survey of data from the National Cancer Database examined 1854 patients with nonfunctioning PNETs < 2 cm. This study found that monitored patients had nearly three times the risk of mortality compared with those who underwent surgical treatment^10^. Meanwhile, the meta-analysis of 714 patients showed that surgical treatment of non-functional PNETs smaller than 2 cm was improved os rates^17^. In our study, the survival analysis showed that surgical treatment could improve overall survival time. However, some studies showed adverse pinons, for example, in a study reviewing 464 patients to observe a patient who has finally developed metastases or died from nonfunctioning PNETs < 3 cm. This study reported that observation was not an increase in death after a median follow-up of 44 months^8^. Another study showed that nonoperative management might be advocated when serial imaging demonstrates minimal or no growth without suspicious features^7^.

In our initial analysis, we identified sex, age, race, and pathological grade as risk factors influencing patient survival. However, even after propensity score matching, age remained a significant risk factor for survival. Traditionally, small non-functional pancreatic neuroendocrine tumors (NF-PNETs) have been considered relatively slow-growing tumors, typically occurring in younger patients, and are generally associated with a favorable prognosis following surgical resection. Our study further demonstrates that younger patients who undergo surgery exhibit the highest survival rates. Pancreatic surgery is a more invasive procedure and is not commonly recommended for older patients. In the surgical group, a higher proportion of younger patients were observed, reflecting the consideration of the surgeon in weighing the benefits and drawbacks of performing surgery in elderly patients, often presenting with symptoms such as tumor obstruction of the pancreatic duct or abdominal pain. Notably, in the older age group, the survival rates were better in the surgery group compared to the observation group. These findings suggest that small non-functioning endocrine tumors may pose a greater risk to long-term survival compared to potential damage to pancreatic function resulting from surgery. Therefore, based on our study, we recommend surgical intervention for small non-functional endocrine tumors to improve patient outcomes.

This study had certain limitations. Firstly, the SEER database lacked specific information on surgical details such as the types of surgeries performed and their timing. Secondly, important factors like Ki-67 and mitotic index, which are critical for tumor classification in the SEER database, were not taken into account in the tumor classification used in this study. Additionally, several significant prognostic factors including disease-free survival, microvascular invasion, vascular resection, R0/R1 resection, anti-tumor immune response, organ specificity, and peptide receptor radionuclide therapy were not considered. The surgical management of neuroendocrine tumors involves various approaches such as radical excision, debulking, and palliative resection, each carrying its own implications. Due to limited data, these procedures were not evaluated in our study, potentially introducing bias. The nature of surgeries also varied, including cases involving obstruction or bleeding, therapeutic excision, palliation, or unmeasured confounding factors, which could introduce bias when using propensity score matching (PSM). Lastly, the SEER database did not provide information on comorbidities, thus precluding an assessment of their impact on overall survival outcomes, which could introduce bias.

## Conclusion

Patients diagnosed with small, non-functioning pancreatic neuroendocrine tumors who undergo surgical intervention exhibit improved overall survival (OS) outcomes. Therefore, surgery is strongly recommended for this patient population.

### Supplementary Information

Supplementary Figure S1.

Supplementary Table S1.

## Data Availability

The datasets used and analyzed during the current study are available from the corresponding author on reasonable request.

## Abbreviations

NF-PNETs: Small non-functional pancreatic neuroendocrine tumors
NCCN: National comprehensive cancer network
ICD-O-3: International classification of diseases for oncology, third edition
SEER: Surveillance, epidemiology, and end results
