# Supplementary material for: Observational study of surgical resection in small non-functional pancreatic neuroendocrine tumors: AS SEER-based study
Source: Sci Rep. 2023 Aug 7;13:12824. doi: 10.1038/s41598-023-39980-z (PMC10406806; doi:10.1038/s41598-023-39980-z)

**Supplement legend**

**Supplement Figure1** Optimal age threshold: (A) Age density distribution plot (B) Maximally Selected Rank Statistics (C) Overall survival analysis in different age group.


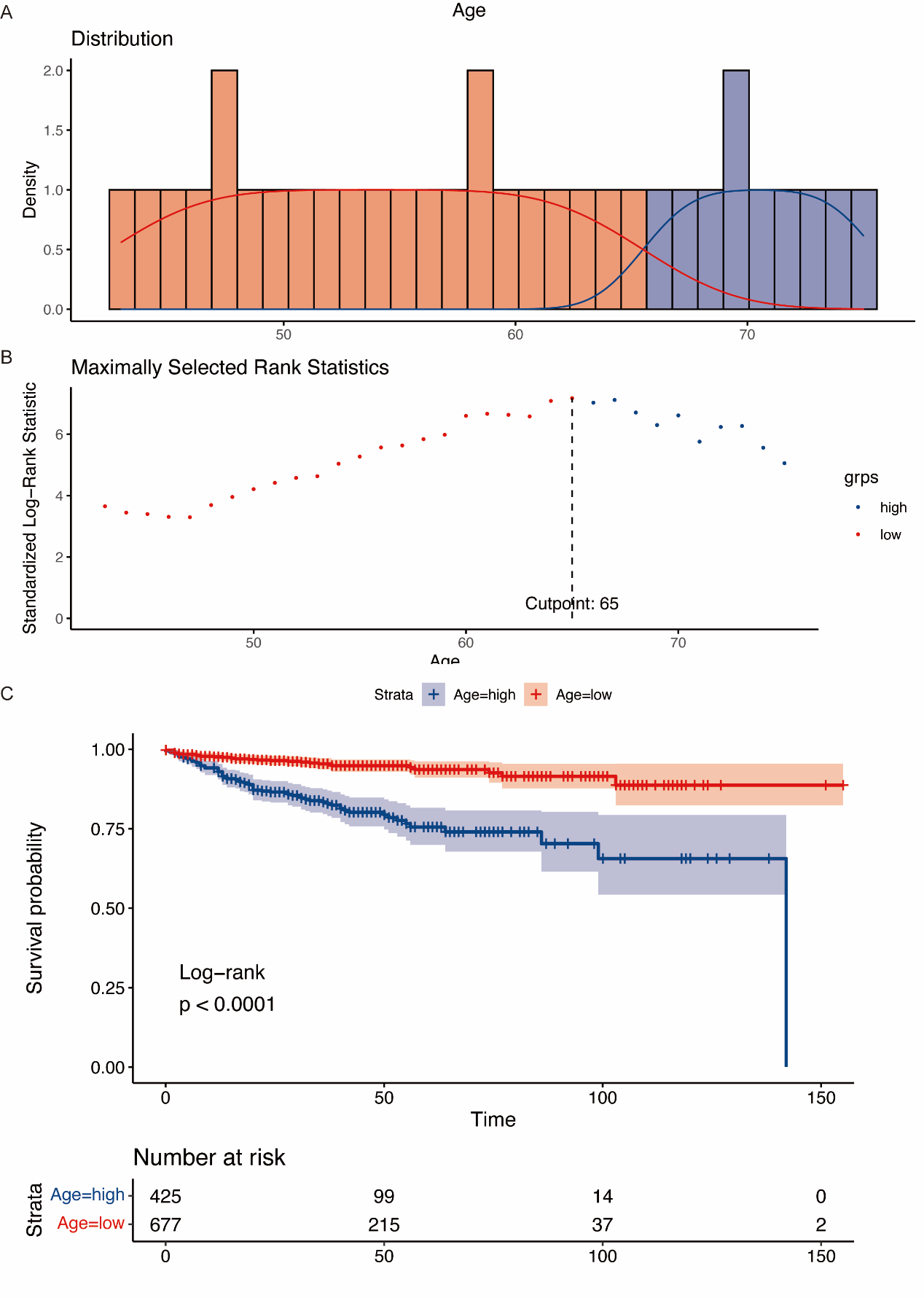

Supplement: Supplementary file 1 — Supplementary Figure S1. [file 41598_2023_39980_MOESM1_ESM.docx]
