# Supplementary material for: Observational study of surgical resection in small non-functional pancreatic neuroendocrine tumors: AS SEER-based study
Source: Sci Rep. 2023 Aug 7;13:12824. doi: 10.1038/s41598-023-39980-z (PMC10406806; doi:10.1038/s41598-023-39980-z)
Supplement: Supplementary file 2 — Supplementary Table S1. [file 41598_2023_39980_MOESM2_ESM.docx]

Supplement Table 1 Results of a sensitivity analysis in matching.

| Gamma | Lower bound | Upper bound |
| --- | --- | --- |
| 1 | 0 | 0.000 |
| 2 | 0 | 0.000 |
| 3 | 0 | 0.00483 |
| 4 | 0 | 0.11721 |
| 5 | 0 | 0.43610 |
| 6 | 0 | 0.73961 |

"Gamma" is a parameter used to quantify the degree of imbalance between the treatment and control groups in propensity score analysis. A Gamma value of 1 indicates perfect balance between the surgical and non-surgical groups in baseline characteristics, indicating no bias. When the Gamma value is greater than 1, it suggests a propensity for the surgical group to receive the treatment, indicating some degree of imbalance. In the case of a Gamma value of 3, it means that the surgical group is three times more likely to receive the treatment compared to the non-surgical group, indicating a significant imbalance. Furthermore, even with a greater likelihood of the surgical group receiving the treatment (Gamma=3), the statistical significance of the treatment effect remains, with an upper bound p-value of 0.0048.
